# *ERG11* Gene Variability and Azole Susceptibility in *Malassezia pachydermatis*

**DOI:** 10.1007/s11046-022-00696-9

**Published:** 2022-12-10

**Authors:** Leyna Díaz, Gemma Castellá, M. Rosa Bragulat, F. Javier Cabañes

**Affiliations:** 1 Veterinary Mycology Group, Department of Animal Health and Anatomy, Universitat Autònoma de Barcelona, Bellaterra, Catalonia Spain; 2 Grup de Micologia Veterinària, Departament de Sanitat i d’Anatomia Animals, Facultat de Veterinària, Universitat Autònoma de Barcelona, 08193 Bellaterra, Barcelona Spain

**Keywords:** Antifungal susceptibility, Azole drugs, *ERG11* mutations, *Malassezia pachydermatis*

## Abstract

*Malassezia pachydermatis* is part of the normal skin microbiota of various animal species but under certain circumstances becomes an opportunistic pathogen producing otitis and dermatitis. Commonly these *Malassezia* diseases are effectively treated using azoles. However, some cases of treatment failure have been reported. Alterations in the *ERG11* gene have been associated with in vitro azole resistance in *M. pachydermatis*. In the present study, in vitro antifungal susceptibility of 89 different strains of *M. pachydermatis* isolated from different animal species and health status was studied. The susceptibility to fluconazole (FLZ), itraconazole (ITZ), ketoconazole and amphotericin B was tested by a disk diffusion method and 17 strains were also subjected to an ITZ E-test. Mueller–Hinton supplemented with 2% glucose and methylene blue was used as culture medium in both susceptibility assays. Multilocus sequence typing was performed in 30 selected strains using D1D2, ITS, CHS2 and β-tubulin genes. Also, *ERG11* gene was sequenced. The four antifungals tested were highly effective against most of the strains. Only two strains showed no inhibition zone to antifungals and a strain showed an increased MIC to ITZ. The study of the *ERG11* sequences revealed a high diversity of DNA sequences and a total of 23 amino acid substitutions, from which only two have been previously described. Also, three deleterious substitutions (A302T, G459D and G461D) previously associated with azole resistance in this yeast were recovered. A correlation between certain genotypes and *ERG11* mutations was observed. Some of the *ERG11* mutations recovered were correlated with a reduced susceptibility to azoles.

**Supplementary Information:**

The online version contains supplementary material available at 10.1007/s11046-022-00696-9.

## Introduction

*Malassezia pachydermatis* is a lipophilic yeast that belongs to the normal skin microbiota of various animal species and colonizes the skin and mucosal sites of healthy dogs. Although *M. pachydermatis* has been considered classically the only non-lipid-dependent species of the genus *Malassezia,* they also lack the genes coding for a fatty acid synthase and thus, are unable to synthetize long-chained fatty acids (C14 or C16) *de* *novo* [1, 2]. *Malassezia pachydermatis* can utilize lipid fractions within the peptone component of SGA, thus they also need a few lipids to be able to grow [2–4].

Despite being part of the microbiota, *Malassezia* may act as an opportunistic pathogen under certain circumstances that allow the overgrowth of the yeast population. *Malassezia pachydermatis* is a common responsible agent of otitis and dermatitis in dogs [5, 6]. In humans, it has also been reported to produce systemic infections particularly in neonates and immunocompromised patients receiving parenteral nutrition [7]. The treatment of *Malassezia* diseases is currently based on the use of topical and systemic antifungal therapy. Topical agents include chlorhexidine, clotrimazole, enilconazole, ketoconazole, miconazole and nystatin that can be used in various forms such as shampoos, sprays, or ointments [8]. Azoles like ketoconazole (KTZ) or itraconazole (ITZ) are commonly used as systemic agents for the treatment of *Malassezia* dermatitis and otitis. Fluconazole (FLZ) can be used for the treatment of *Malassezia* skin diseases like seborrheic dermatitis [9, 10]. Amphotericin B (AMB) and liposomal amphotericin B are indicated in the treatment of systemic cases of *Malassezia* in humans [8, 11].

The Clinical and Laboratory Standards Institute (CLSI) developed in 2002 a reference broth microdilution method for evaluating the susceptibility of *Candida* spp. and *Cryptococcus neoformans* [12]. This method is inapplicable to the genus *Malassezia* because of their lipid-dependency, their slower growth rate and tendency to form clusters [13, 14]. Thus, it has been subsequently adapted to *Malassezia* by various researchers modifying the media, time of incubation and inoculum but it has not been yet standardized [13, 15–17]. Also, disk diffusion method and the E-test gradient have been adapted to evaluate the susceptibility of *Malassezia* against antifungal compounds [14, 18].

Although antimicrobial resistance is a global serious threat to human and animal health, recent studies have showed that most wild-type *Malassezia* yeast remain susceptible to the most used azoles such as KTZ, ITZ and miconazole but the efficacy of FLZ is variable [8, 11, 19]. Some sporadic reports of therapeutic failure with azoles in canine *M. pachydermatis* dermatitis associated with increased tolerance to azoles in vitro might reflect the chronic and relapsing course of *Malassezia* diseases that often need frequent and lengthy treatments [16, 20–23].

There are various mechanisms of azole antifungal resistance in *Malassezia* species. The drug efflux pump is associated to azole resistance in *M. pachydermatis* [14, 25]. Mutations in the coding region of the *ERG11* gene, encoding a lanosterol 14α-demethylase, which is the target enzyme of azole antifungals, are considered a mechanism of resistance [26–28]. Kim et al. [29] demonstrated that tandem quadruplication of the genomic region of the *ERG11* gene contributes to azole resistance in *M. pachydermatis*. At the present, only two *M. pachydermatis* strains have demonstrated to be resistant to azoles. The strains were isolated in Japan [23] and Italy [22]. Both isolates showed high minimum inhibitory concentrations (MICs) against azoles but only in one case this resistance was linked to *ERG11* mutations [23]. Although some mutations have been detected in this gene, its intrinsic diversity in *M. pachydermatis* yeasts, that are mainly susceptible to antifungals, is yet unknown.

Even though there is not a reference method for susceptibility testing of *M. pachydermatis* available yet, on this study we evaluate the susceptibility of different strains of this yeast against three different azoles (FLZ, ITZ, and KTZ) and AMB. We included a wide variety of strains recovered from different animal species with different health status and collected over different years. Also, we study the genetic variability of *ERG11* gene in these strains. Gene mutations and antifungal susceptibility testing results was investigated.

## Materials and Methods

### Strains

A total of 89 strains of *M. pachydermatis* (Table S1) were selected from our culture collection for susceptibility testing. Strains were isolated between 1994 and 2021 and included different animal species: dog (n = 73), cat (n = 12), pig (n = 1), cow (n = 1), goat (n = 1), and horse (n = 1). The *M. pachydermatis* neotype strain CBS1879 was also included. The strains were stored at − 80 ºC [24].

The 73 strains of dogs included four strains isolated from healthy dogs, eight strains from dogs with dermatitis, 59 from dogs with otitis externa and two from otitis media. According to the data provided by the clinicians when sending the samples, the strains from otitis externa were classified into chronic otitis externa (lasting more than three months, n = 25), acute otitis externa (lasting less than six weeks, n = 17), purulent otitis externa (n = 6) and recurrent otitis externa (n = 4). The strains isolated from cats included five strains recovered from cats with dermatitis and six strains from animals with otitis, from which two were otitis media and four otitis externa. The strains from otitis externa were classified into acute otitis externa (n = 2), chronic otitis externa (n = 1) and purulent otitis externa (n = 1). One strain from a healthy cat was also included. The strains from cow, horse, pig, and goat were all obtained from healthy animals.

### Susceptibility Testing

Four different antifungals were selected for susceptibility testing: amphotericin B (AMB), fluconazole (FLZ), itraconazole (ITZ) and ketoconazole (KTZ). Susceptibility of the strains was tested by a disk diffusion method. For the disk diffusion method, two stock inoculum suspensions were prepared from 3-day old cultures on SGA (Oxoid) at 35ºC from each strain. The stock inoculum suspensions were performed in 3 mL of distilled water supplemented with 0.004% Tween 80 to reduce clump formation and were adjusted to a density of 1 McFarland standards (3 × 10^8^ CFU/ml). Mueller–Hinton supplemented with 2% glucose and 0.5 mg/l methylene blue (MH-GM) was the media selected for the disk diffusion method [13, 30]. For each suspension tube, 5 plates of the media were streaked using a sterile cotton swab dipped into the inoculum suspension. One of the plates was a growth control and on the remaining four, one disk of antifungal per plate was placed. Two commercial antifungal disks were used comprising AMB, FLZ, ITZ and KTZ (Neo-Sensitabs, Rosco) at concentrations of 10, 25, 10 and 15 µg/disk respectively and FLZ, KTZ and AMB (Bio-Rad) at concentrations of 25, 50 and 100 µg/disk, respectively. Plates were then incubated at 35ºC, and inhibition zone sizes were measured at 48, 72 and 96 h. The inhibition zone diameter was determined after incubating at 35ºC for 72 h. *Candida parapsilopsis* ATCC 90,028 and *Pichia kundriavzevii* ATCC 6258 were included as quality control strains in both disk diffusion tests.

The interpretative criterion was according to the manufacturer’s guidelines for yeasts, as shown in Table 1.

**Table 1:** Interpretative criterion of zones of inhibition

Inhibitory zone diameter (mm)						
	Rosco©			Bio-Rad©		
	S	SDD	R	S	SDD	R
Amphotericin B	≥ 15	14–10	≤ 10	> 10	–	≤ 10
Fluconazole	≥ 19	18–15	≤ 14	≥ 19	18–15	≤ 14
Itraconazole	≥ 23	22–14	≤ 13	–	–	–
Ketoconazole	≥ 28	27–21	≤ 20	≥ 20	20–10	≤ 10

S, susceptible; SDD, susceptible dose dependent; R, resistant

(–) No Data

Susceptibility against ITZ was also studied using the E-test method on 17 out of the 89 strains. The 17 strains were selected based on their diverse response to antifungals by disk diffusion method, but also different animal species were included. The strain selection included strains obtained from healthy animals (n = 7), from animals with otitis externa (n = 8) and animals with dermatitis (n = 2). Also, the *M. pachydermatis* neotype strain CBS1879 was included. E-test was performed under the same conditions that the disk diffusion method. One E-test gradient stripe for ITZ (Biomérieux) was placed on each plate. The plates were then incubated at 35ºC and examined at 48, 72 and 96 h. The MICs were determined after 72 h of incubation. Also, some strains were evaluated using SGA supplemented with 1% Tween 80 [23] to compare the results obtained.

### *ERG11* Gene Amplification and Sequencing

A total of 31 strains were selected to sequence their *ERG11* gene, including the neotype strain of *M. pachydermatis* CBS1879 and the 17 strains evaluated by E-test. The strains selected included different animal species, dog (n = 23), cat (n = 4), pig (n = 1), horse (n = 1), goat (n = 1) and cow (n = 1). These strains were recovered from nine healthy animals, 17 animals with otitis externa, one animal with otitis media and four animals with dermatitis. The strains were selected based on their susceptibility results against the four antifungals tested. Strains without an inhibition zone, a reduced inhibition zone in the disk diffusion method and a high MIC to ITZ were included. Also, strains with wider inhibition zone diameters were included.

The DNA was extracted directly from 5-day-old cultures grown in SGA according to the FastDNA Spin kit protocol (MP Bio-medicals, Biolink, Barcelona, Spain). The *ERG11* gene was amplified using the primers pairs MALAERG1S/R, MALAERG2S/R, MALAERG3S/R, MALAERG4S/R described by Kano et al. [23]. Reaction mixtures contained 5.0 μl of template DNA, 5.0 μl of 10 × PCR buffer, 0.2 mM of each dNTP, 1.5 mM MgCl2, 0.2 μM each primer and 1.25 U of *Taq* polymerase in a final volume of 50 μl. The amplification process consisted of a pre-denaturation step at 95 ºC for 10 min, followed by 35 cycles of denaturation at 95 ºC /15 s, annealing at 55 ºC /30 s and extension at 72 ºC /1 min, and a final extension of 5 min at 72 ºC. Both strands of purified gene fragments were sequenced with BigDye Terminator v3.1. cycle sequencing kit (Applied Biosystems) on an Applied Biosystems 3730 DNA Analyzer (Applied Biosystems). Sequence alignments were carried out using Clustal X v2.0.12. software [31]. The PROVEAN software [32] was used to study the effect on the protein of the amino acid substitutions.

Maximum likelihood analysis of the *ERG11* sequences was conducted using MEGA 11 software [33] with 1,000 bootstrap replicates. Clades that were supported by bootstrap values of ≥ 70% were regarded as strongly supported.

### Multilocus Sequences Typing

The strains selected for *ERG11* gene sequencing were also chosen for a multilocus sequence typing system based on four target regions (D1/D2 region of the 26S rRNA gene, the ITS-5.8S rRNA gene, the β-tubulin gene and the CHS2 gene).

Sequences of the four genes of 16 strains had been characterized previously [34]. The four genes of 15 remaining strains were amplified and sequenced as described previously [35]. To illustrate the phylogenetic relatedness between our strains a minimum spanning tree was constructed using the PHYLOViZ 2.0 software [36]. Each genotype cluster was identified by using the goeBURST algorithm version.

### Statistical Analysis

Statistical analyses were conducted by Minitab 17 Statistical software (Minitab). The results of both dilutions of each strain were compared using the Student’s t-test. To compare the means of inhibition zone diameter between strains obtained from healthy animals and strains obtained from animals with dermatitis/otitis the ANOVA test was used.

## Results

### Susceptibility Testing

All the strains tested showed growth on the media selected (MH-GM). Susceptibility results were determined after 72 h of incubation at 35ºC, since it was the optimal time of growth for all the strains included. At 48 h not all the strains had growth and no differences were observed between inhibition zone diameters at 72 and 96 h. The results of both disk diffusion methods at 72 h are summarized in Table 2. No significant differences were observed between both dilutions of each strain (*P* > 0.05).

**Table 2:** Mean (± SD) inhibition diameters at 72 h and range to fluconazole (FLZ), itraconazole (ITZ), ketoconazole (KTZ) and amphotericin B (AMB) by both disk diffusion tests performed in strains from healthy animals, animals with otitis and animals with dermatitis

	HEALTHY (n = 9)			OTITIS (n = 67)			DERMATITIS (n = 13)			TOTAL (n = 89)			*p* value
	n	X ± SD	range	n	X ± SD	range	n	X ± SD	range	n	X ± SD	range	
FLZ													
R 25 µg	4	32.25 ± 2.90	29.00–36.00	67	30.50 ± 10.31	7.50–56.50	12	30.38 ± 11.76	0- 42.00	83	30.57 ± 10.22	0–56.50	0.945
B 25 µg	8	35.19 ± 3.94	28.00–39.00	66	33.68 ± 12.82	5–73.00	12	35.75 ± 17.06	0- 68.00	86	34.11 ± 12.49	0–73.00	0.775
ITZ													
R 10 µg	8	36.44 ± 3.97	29.50–42.00	67	39.43 ± 6.80	19.0–58.00	12	39.88 ± 5.41	28.00–47.50	87	39.22 ± 6.43	19.00– 58.00	0.433
KTZ													
R 15 µg	8	48.94 ± 2.80	44.00–53.00	67	53.78 ± 7.88	20.00–73.00	12	53.71 ± 3.86	45.50–59.00	87	53.32 ± 7.22	20.00–73.00	0.199
B 50 µg	8	56.94 ± 3.45	53.00–63.00	67	62.79 ± 6.45	38.50–78.00	13	66.85 ± 5.12	57.50–77.00	88	62.86 ± 6.46	38.50–78.00	0.002
AMB													
R 10 µg	4	16.63 ± 0.75	15.50–17.00	67	17.33 ± 2.99	0–22.00	12	17.88 ± 1.55	15.00–20.00	83	17.37 ± 2.76	0–22.00	0.706
B 100 µg	8	19.88 ± 1.22	18.00–22.00	67	19.88 ± 2.73	13.00–27.50	13	19.46 ± 2.20	16.00–23.00	88	19.82 ± 2.54	13.00–27.50	0.530

R, Rosco©; B, Bio-Rad©

In general, according to the manufacturer’s breakpoints for yeasts, the four antifungals tested were active against most of the strains regardless of their origin. However, a few exceptions were observed. With Rosco antifungal disks, five strains were classified as susceptible dose dependent (SDD) and five as resistant (R) to FLZ. One strain was classified as R to KTZ, one as SDD to ITZ, and four as SDD and one as R to AMB. One strain (MA165) was classified as R to ITZ, KTZ and AMB, and one strain (MA1386) was classified as R to FLZ and SDD to AMB. With Bio-Rad antifungal disks, four strains were classified as SDD and seven as R to FLZ. These strains were recovered from animals with chronic otitis externa or dermatitis. All strains recovered from healthy animals were classified as susceptible according to the manufacturer’s breakpoints for yeasts. Strain MA1429, isolated from a dog with dermatitis, showed the complete absence of inhibition zone to FLZ and strain MA165, isolated from a dog with chronic otitis externa, showed no inhibition zone to AMB.

No differences were observed in the inhibition zone diameters between strains recovered from healthy animals and strains from animals with otitis or dermatitis except for the KTZ Bio-Rad antifungal disks (Table 2). Strains recovered from chronic otitis externa and otitis media showed smaller inhibition diameters to KTZ than strains from purulent and acute otitis externa (*P* = 0.010).

**Fig. 1:**
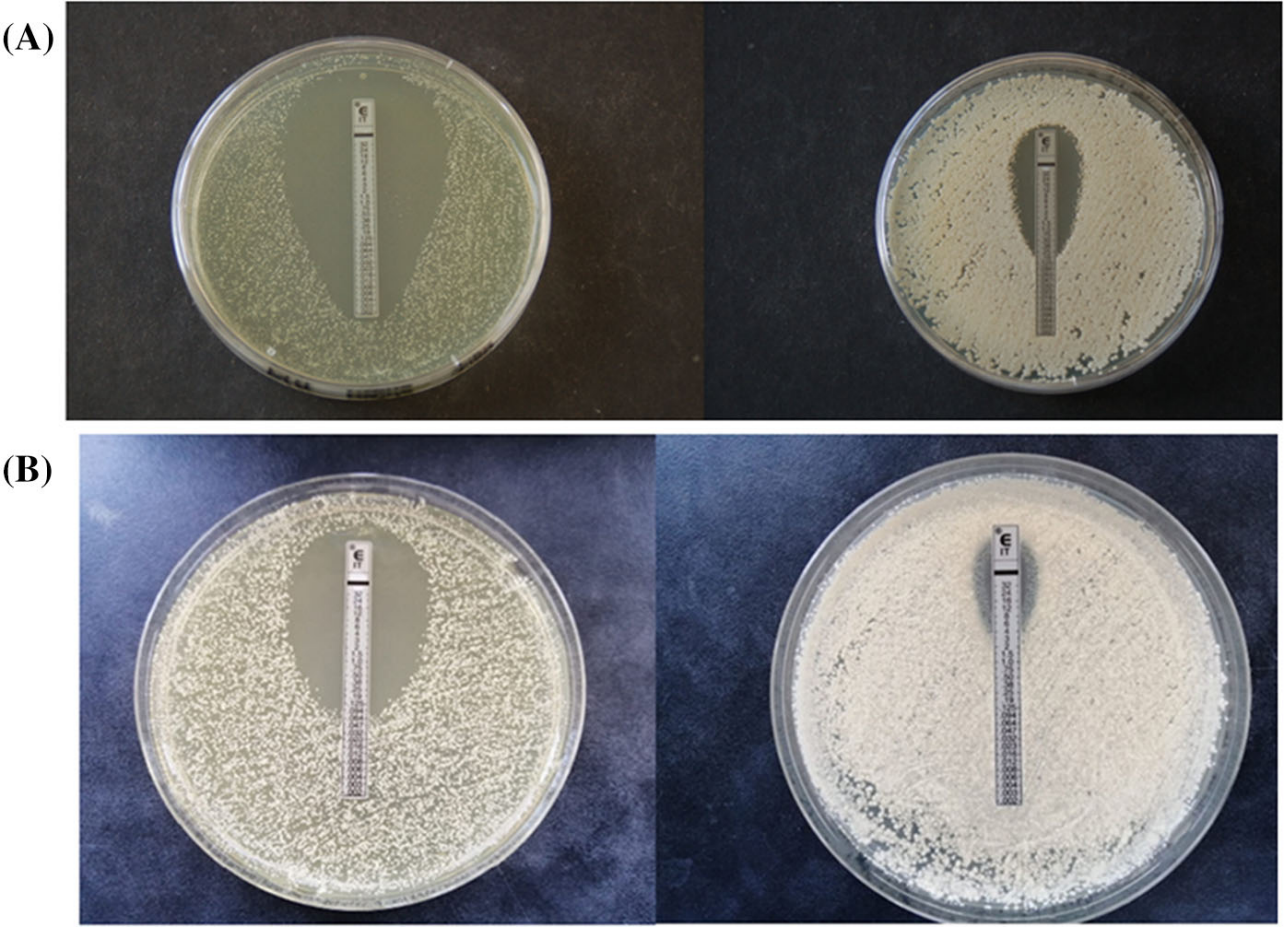
E-test assay of two isolates of *M. pachydermatis* against itraconazole. A) Reference strain CBS1879 in MH-GM (left) and SGA + Tween 80 (right) culture media. B) Strain MA1716 in MH-GM (left) and SGA + Tween 80 (right) culture media

Most of the strains selected for the E-test method using MH-GM as culture medium, showed MICs between 0.002 and 0.003 µg/ml to ITZ (Table 3). The only exception was strain MA1716 that showed a MIC of 0.125 µg/ml (Fig. 1). This strain was also tested using SGA supplemented with 1% Tween 80 and a MIC of 8-12 µg/ml was obtained. The neotype strain CBS 1879 showed a MIC of 0.002 µg/ml and 0.38 µg/ml in MH-GM and SGA + 1% Tween 80, respectively.

**Table 3:** Strains of *Malassezia pachydermatis* used, including animal host, health status, corresponding type of *ERG11* sequence, amino acid substitutions, mean inhibition diameters of the disk diffusion method and itraconazole minimum inhibitory concentration (MIC) using E-test method at 72 h

Strain	Host	Health status	*ERG11* DNA Sequence type	Amino acid substitutions	Mean inhibition diameters (mm) values at 72 h					MIC (µg/ml)
					FLZ (R)	FLZ (B)	ITZ (R)	KTZ (R)	KTZ (B)	E-test ITZ
CBS1879	Dog	OE	I^a^	**–**	28	36.5	37.5	49	54.5	0.002
CBS6535	Dog	H	I^a^	**–**	26	36.5	34	50	55	0.003
MA13	Dog	H	I^a^	**–**	36	36	40	53	63	0.003
MA52	Dog	H	I^a^	**–**	34	37	38.5	51.5	58.5	0.002
MA56	Dog	H	I^a^	**–**	35	35.5	38	47.5	59.5	0.003
MA195	Dog	OE	I^a^	**–**	56.5	47	57	64	63.5	0.002
MA1595	Cow	H	I^a^	**–**	36	39	35	49.5	57	–
CBS1884	Dog	OE	II^a^	E181Q	35	42.5	42	51.5	69.5	0.002
MA94	Horse	H	II^a^	E181Q	31.5	39	34.5	49	53	0.002
MA312	Cat	OE	XI^a^	E181Q	36	38.5	37.5	48.5	62	0.003
MA587	Cat	OM	II^a^	E181Q	37.5	54.5	36.5	46.5	38.5	–
MA579	Cat	D	XVIII^a^	I25V, E181Q	31.5	41	40	54	67	0.002
MA1716	Dog	D	XXI^a^	**A302T, G459D**	–	6.5	–	–	59	0.125
MA10	Dog	OE (C)	VIII^a^	I25V, E181Q, T354I	31	28	58	47	49	–
MA140	Cat	H	IV^a^	I25V, E181Q, T354I	32.5	28	42	47	56	0.002
MA1401	Dog	OE (R)	XVI^a^	V33I, E181Q, **R202H**	23	30	34	45	60	–
MA475	Pig	H	V^b^	R84K, D166E, E181Q, D405N	29	30.5	29.5	44	53	0.002
MA7	Dog	OE (R)	VI^b^	A17T, R84K, R175H, Q178R, E181Q	21	17.5	30	50	61	–
MA8	Dog	OE (C)	VII^b^	A17T, R84K, R175H, Q178R, E181Q	40.5	41.5	45.5	59	60	–
MA856	Dog	OE (A)	XIV^b^	A17T, R84K, R175H, Q178R, E181Q	20	24	33	39.5	63.5	–
MA944	Dog	OE (C)	XIV^b^	A17T, R84K, R175H, Q178R, E181Q	15.5	27.5	29	45	59	–
MA968	Dog	OE (P)	XIV^b^	A17T, R84K, R175H, Q178R, E181Q	25	25	25	44.5	68.5	–
MA1382	Dog	OE (C)	XV^b^	A17T, R84K, R175H, Q178R, E181Q	16	18.5	41	54	59.5	0.002
MA107	Goat	H	III^c^	E181Q, N212S, E290D, **Y352F**, H399R	–	–	–	–	–	–
MA165	Dog	OE (C)	IX^b^	A17T, R84K, **F143S**, R175H, Q178R, E181Q	21	33.5	19	20	66.5	0.002
MA356	Dog	O	XII^b^	A17T, R84K, Q178R, E181Q, **Y352F**, H399R	23.5	27.5	39	51	53.5	0.002
MA280	Dog	OE (C)	X^c^	I25S, **W52L**, R84K, L86F, E181Q, N212S, E290D, **Y352F**, H399R	24.5	9.5	46	62.5	64	0.002
MA361	Dog	OE (C)	XIII^c^	I25S, **W52L**, R84K, L86F, E181Q, N212S, E290D, **Y352F**, H399R	18	20	34	51	63	–
MA1289	Dog	D	XIX^c^	I25S, **W52L**, R84K, L86F, E181Q, N212S, E290D, **Y352F**, H399R	19	19	40	52.5	65	–
MA1478	Dog	OE (A)	XVII^c^	I25S, **W52L**, R84K, L86F, E181Q, N212S, **S226L**, E290D, **Y352F**, H399R	8	5.5	32	59	58.5	–
MA1429	Dog	D	XX^c^	I25S, **W52L**, R84K, L86F, E181Q, N212S, E290D, **A306S, Y352F**, H399R, **G461D**	0	0	32.5	47	64.5	–

In bold, deleterious mutations

OE, otitis externa; OM, otitis media; A, acute; C, chronic; P, purulent; R, recurrent; H, healthy; D, dermatitis; FLZ, fluconazole; ITZ, itraconazole; KTZ, ketoconazole; R, Rosco©; B, Bio-Rad©

Subclades from the phylogenetic analysis of *ERG11* gene: ^a^subclade I; ^b^subclade III; ^c^subclade II

– Not tested

### *ERG11* Sequence

Amplification of the complete *ERG11* gene and screening for amino acid substitutions was performed for each of the 31 strains (Table 3). The *ERG11* sequence included 1623 bp. The sequences of the strains shared 96.0–100% sequence similarity to *M. pachydermatis* neotype strain CBS1879 and were clustered into 21 different genetic types. Representative nucleotide sequences of the different genetic types determined in this study have been deposited at the GenBank database under accession numbers ON814677- ON814697.

Of the 31 strains of *M. pachydermatis*, seven of them belonged to the genetic type I, including the neotype strain CBS1879. All strains of genetic type I were isolated from healthy animals except two (MA195 and CBS1879). Genetic type II included three strains recovered from different animal species and genetic type XIV included three strains recovered from dogs with otitis. The rest of genetic types were unique for each strain.

The predicted *ERG11* amino acid sequences consisted of 540 amino acids. Some silent polymorphisms were identified, and comparison of the deduced amino acid sequences revealed 14 unique amino acid sequence types. Strains belonging to genetic type I had no amino acid substitution. Strains from genetic type II and XI showed the same amino acid sequence with one amino acid substitution (E181Q). Strains from genetic type IV and VIII showed the same amino acid sequence with three substitutions (I25V, E181Q, and T354I). Strains from genetic type X, XIII and XIX showed the same amino acid sequence with nine substitutions (I25S, W52L, R84K, L86F, E181Q, N212S, S226L, E290D, Y352F, H399R). Strains from genetic type VI, VII, XIV and XV showed the same amino acid sequence with five substitutions (A17T, R84K, R175H, Q178R, and E181Q). The number of amino acid substitutions of the rest of genetic types varied between the strains and ranged from 2 to 11 substitutions per strain. A total of 23 amino acid substitutions were identified. Fourteen amino acid substitutions were considered as neutral: A17T, I25V, I25S, V33I, R84K, L86F, D166E, R175H, Q178R, E181Q, N212S, E290D, T354I, H399R, D405N. All these mutations were recovered from strains isolated from different animal species and health status. Nine amino acid substitutions were considered as deleterious substitutions (W52L, F143S, R202H, S226L, A302T, A306S, Y352F, G459D, G461D) and were recovered from strains isolated mainly from dogs with otitis or dermatitis. Mutations A302T, G459D were detected in strain MA1716 with a MIC of 0.125 µg/ml and mutations W52L, A306S, Y352F, and G461D were detected in strain MA1429 with no inhibition zone to FLZ.

**Fig. 2:**
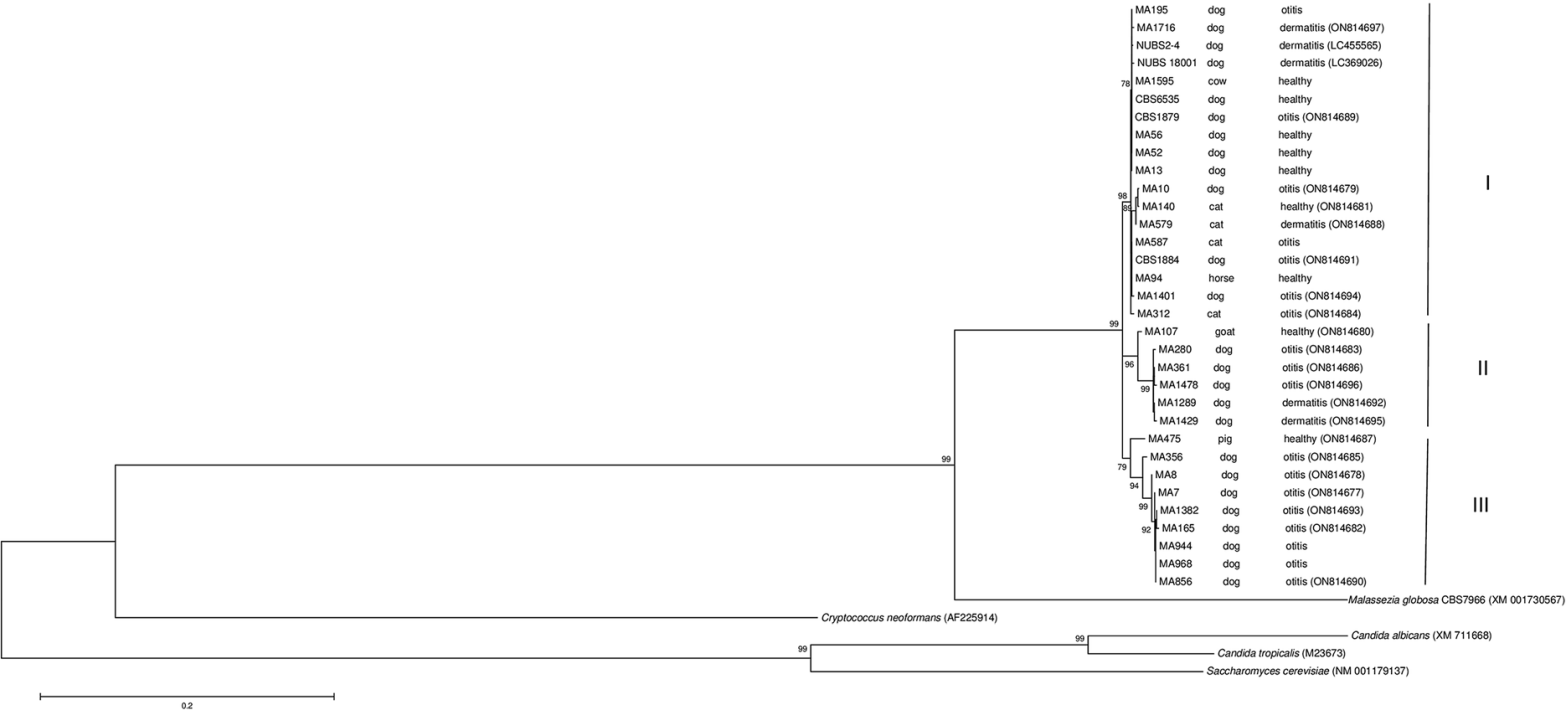
Molecular phylogenetic tree inferred from maximum likelihood analysis of *ERG11* sequences of *M. pachydermatis* strains. Bootstrap values > 70% in 1,000 replications are shown at the nodes. Sequences of *Cryptococcus neoformans*, *Candida albicans*, *Candida tropicalis* and *Saccharomyces cerevisiae* were selected as outgroup for the tree construction

Maximum likelihood analysis of the *ERG11* gene is shown in Fig. 2. All *M. pachydermatis* sequences grouped in a supported clade. The strains were grouped in three different subclades. Subclade I (94% bootstrap) grouped strains from healthy animals and animals with otitis or dermatitis. Subclade II (96% bootstrap) grouped mainly strains isolated from dogs with otitis and dermatitis and one strain from a healthy goat. A third subclade, subclade III (81% bootstrap) grouped strains isolated from dogs with otitis and one strain from a healthy pig.

### Multilocus Sequence Typing

Sequence types obtained for each gene are listed in Supplementary Table S2. Six different sequence types of D1/D2 region were recovered, five from them were previously described and one is new from this study (VI). Eleven out of the 30 strains (36.66%) had sequence type I which was the most abundant. Strains from healthy animals only showed sequence types I or II.

The ITS 5.8S rRNA was successfully amplified and sequenced. Fifteen different sequence types were recovered from which four were new (XIV, XV, XVI and XVII). Ten strains had one of the new ITS sequence types. The sequence type I was the majority (n = 5; 16.6%) and was recovered from strains from dogs.

Ten different sequence types of β-tubulin were recovered from which two were new ones (X and XI). Sequence type VIII was the most abundant and was recovered from eight strains (26%) isolated from dogs with otitis or dermatitis.

Regarding CHS2 gene, nine different sequence types were recovered in total, and all of them have been described previously. Sequence type I was majority and was obtained from a total of nine strains (30%) of dogs (n = 6), horse (n = 1), cat (n = 1) and cow (n = 1).

The sequences obtained in this study have been deposited at the GenBank database under accession numbers ON787824 (D1D2), ON791562-ON791565 (ITS), and ON814675- ON814676 (β-tubulin).

**Fig. 3:**
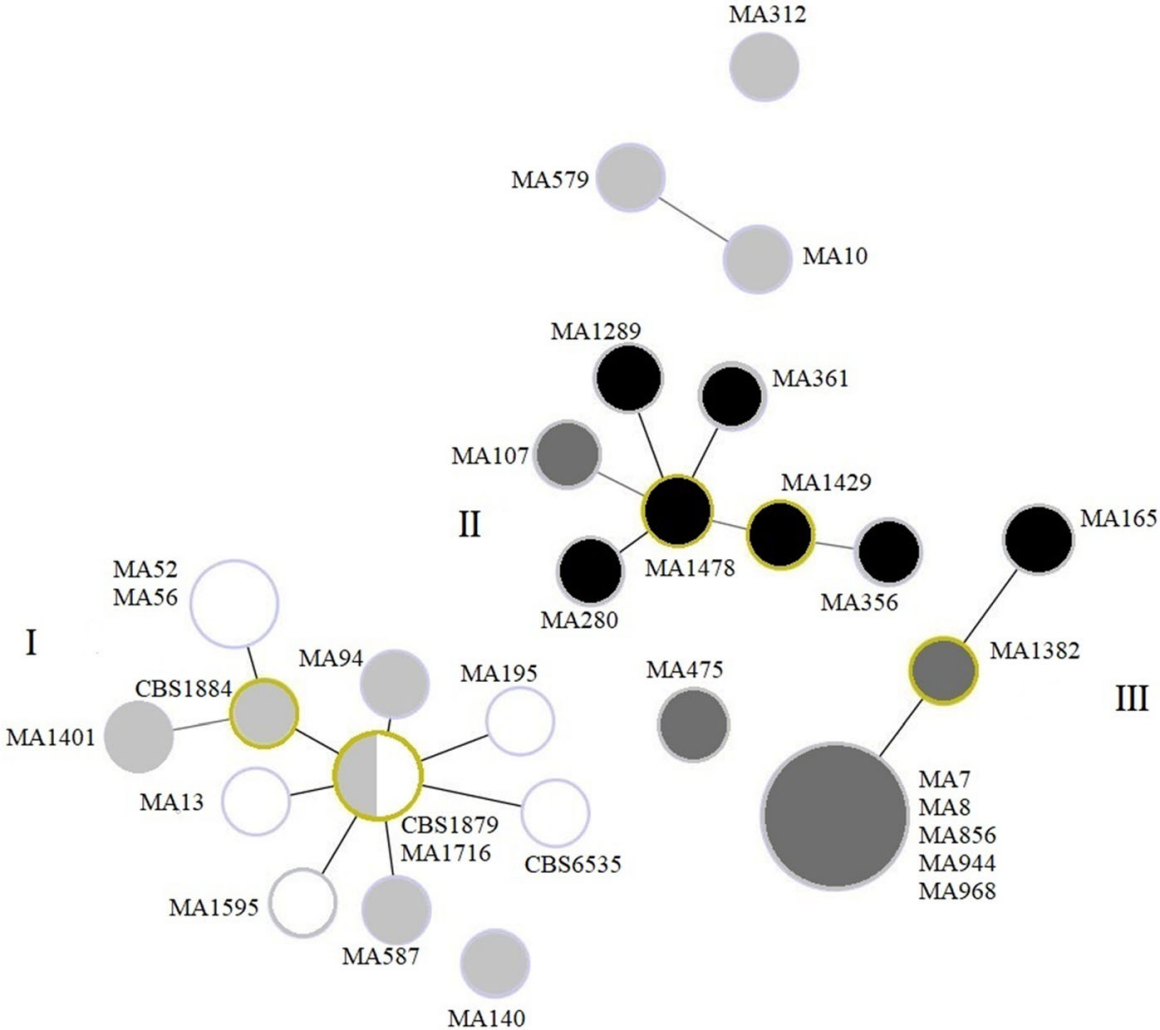
Minimum spanning trees using the goeBURST algorithm of 31 *Malassezia pachydermatis* strains based on analysis of the four loci used for genotyping. Each circle represents a unique genotype. The diameter of each circle corresponds to the number of isolates sharing the same genotype and the numbers within circles represent strain number. The color of the circle is related to the presence of amino acid substitutions in *ERG11*: black for strains with 6 to 11 amino acid substitutions, dark gray for strains with 4 to 5 amino acid substitutions, light gray for strains with 1 to 3 amino acid substitutions and white for strains without any amino acid substitution

When the four loci (D1D2, ITS, CHS2 and β-tubulin) were combined, a total of 25 genotypes were identified. Among all genotypes, 22 were only found once. Two genotypes were shared by two strains, and one genotype was shared by five strains. When the presence of amino acid substitutions was considered, the graphing algorithm analysis revealed small clusters of *ERG11*-mutated isolates. As shown in Fig. 3, a cluster of 12 strains of 10 different genotypes (I) with a few amino acid substitutions (0–3) was observed. All the strains within this group showed wide inhibition diameters to azoles except for one strain (MA1716) with a small inhibition diameter to FLZ and a higher MIC to ITZ. A second cluster (II) included seven strains of seven different genotypes with five to 11 amino acid mutations. One strain within this cluster (MA1429) showed no inhibition zone to FLZ. Also, a third cluster (III) of seven strains of three different genotypes was obtained with five to six amino acid substitutions. One strain was considered as R to KTZ and SDD to ITZ, and two strains were considered SDD to FLZ.

## Discussion

Antifungal susceptibility testing for *M. pachydermatis* must be interpreted with caution neither breakpoints nor a reference method have been yet stablished for this yeast species. This means that any classification of the strains into susceptible, intermediate, and resistant remains speculative. However, according to the manufacturer’s breakpoints available for yeasts the four antifungals tested in our study were active against most of the strains selected. Only one strain in our study showed an increased MIC to ITZ. As reported by some authors [8, 16, 37, 38], *M. pachydermatis* is highly susceptible to KTZ and ITZ which is consistent with the results observed in our study. Since now, only two *M. pachydermatis* isolates have demonstrated to be resistant to both ITZ and KTZ [22, 23]. In our study, only one strain showed no inhibition zone to FLZ. Fluconazole consistently returns significantly higher MICs when compared to other antifungals tested among studies with *M. pachydermatis* [17, 39, 40]. It has been demonstrated that *M. pachydermatis* isolates can become resistant during treatment with FLZ by inducing resistance in vitro to this antifungal [41]. Thus, the clinical utility of this azole in dogs and cats is questionable [8, 42]. Bernardo et al. [43] found a *M. pachydermatis* isolate resistant to AMB. However, due to the potential toxicity of AMB its use in veterinary medicine is limited to serious progressive or disseminated systemic mycoses [8].

In our study, all the strains with a reduced inhibition zone or no inhibition zone were recovered from animals with otitis and/or dermatitis. These results agree with the ones obtained in three different studies where the MICs of various antifungals agents were higher for isolates from animals with otitis/dermatitis [21, 38, 44]. Also, in our study, differences were observed in the inhibition diameters between strains from chronic otitis externa and otitis media, and strains from purulent and acute otitis externa. The mean inhibition diameters to KTZ were higher in the strains from acute and purulent otitis externa. A study by Chiavassa et al. [20] compared the MIC values of two different antifungal agents between isolates of *M. pachydermatis* from chronic otitis and acute otitis externa. The results showed that the isolates from chronic otitis externa had MIC values higher than the isolates from acute otitis externa. It was hypothesized that those increased MICs were a result to the exposure of isolates to antifungal agents. In our study, strains MA1716, with an increased MIC to ITZ, and MA1429, with complete absence of inhibition zone to FLZ, were recovered from dogs with previous antifungal treatments with ITZ and miconazole, respectively.

In the E-test method, only one strain (MA1716) showed higher MICs in MH-GM and SGA + 1% Tween 80 and thus, could be considered to have a reduced susceptibility against ITZ. An increase of 64-fold of the MIC was observed when the media used was SGA with lipid supplementation. This increase in MIC values when the culture medium has a lipid supplementation was also observed in *M. pachydermatis* CBS1879 (Fig. 1). This medium was also used to assess susceptibility to ITZ and KTZ by the E-test technique [23]. Different testing variables are known to have an impact on in vitro determinations as lipid supplementation enhancing the yeast growth [8, 45]. Thus, the isolates could appear to be susceptible or resistant only by modifying test conditions. Due to this, it is essential to establish a set of standardized criteria for in vitro susceptibility testing of *Malassezia* spp. [14, 46].

Mutations of the *ERG11* gene could reduce the susceptibility of fungi to azoles [26–28, 47–49]. In our study, a high variability of this gene was observed. Sequence differences in the *ERG11* gene among strains ranged from 0.1 to 4.0%, which is greater than those described with CHS2 (1.9 to 3.4%) or β-tubulin (0.3 to 3.4%) [34]. Indeed, only 6 of the strains displayed the sequence of the *M. pachydermatis* neotype strain CBS1879. This strain has been previously used as comparison to detect possible amino acid mutations [23]. The analysis of the *ERG11* gene grouped *M. pachydermatis* strains in three different clades. This aggrupation correlates quite well with the groups defined by multilocus sequencing.

Even though many polymorphisms were observed, not all lead into amino acid substitutions. Our data clearly show that in *M. pachydermatis* point mutations leading to amino acid changes are a frequent event in *ERG11* gene. A total of 23 different amino acid substitutions were recovered from which nine were deleterious substitutions. The analysis of multilocus groups using *ERG11* amino acid substitutions as discriminant parameter revealed some closely related genotypes carrying more amino acid substitutions. This suggested a correlation between certain genotypes of *M. pachydermatis* and in vitro susceptibility results, in agreement with other authors [38, 50].

Of the amino acid substitutions identified, only two (G459D, G461D) have been reported previously in *M. pachydermatis* associated with azole resistance [51, 52]. One of the mutations present in our study, G461D, recovered from a strain with no inhibition zone to FLZ, was also observed in a clinical isolate with in vitro resistance to ravuconazole [51].

The other mutation, G459D, was reported in miconazole tolerant clones of the CBS1879, selected by serial passage on miconazole supplemented media [52]. This mutation was also present in a strain (MA1716) with a higher MIC to ITZ and with a reduced inhibition zone to FLZ in our study. This strain also showed a mutation at point A302T. This point mutation but with a different amino acid change (A302V) has been described by Kano et al. [23] in an isolate with proven in vitro resistance to ITZ and KTZ. The different amino acid change could explain the results obtained in susceptibility testing of our strain. Another point mutation described by Kano et al. [23] and associated with azole resistance, M138V, was not detected in our study.

Point mutations of the *ERG11* gene leading to amino acid substitutions that induce antifungal resistance have also been observed in other *Malassezia* species [47, 53]. In *M. furfur* a point mutation Y67F (130 in *M. pachydermatis*) is associated with fluconazole resistance [53]. In *M. globosa* three-point mutations Y127F, A169S and K176N (synonymous with 130, 172 and 179 in *M. pachydermatis*) are associated with azole resistance [47]. However, none of these point mutations were observed in the strains of this study.

Some of the amino acid substitutions observed in our study had been described in other fungal species associated to a reduced antifungal susceptibility. In *Candida albicans*, point mutations at positions 54, 145, 226 and 307 (synonymous with 52, 143, 202, and 226 in *M. pachydermatis*, respectively) were associated with a reduced azole susceptibility [54–57]. These point mutations are deleterious in *M. pachydermatis* and were observed in our study in some strains. Also, the presence of two amino acid substitutions in combination G307S + G450E in *C. albicans* was reported to increase by 16-fold the MICs to FLZ [56]. This combination is synonymous with the combination A306S + G461D in *M. pachydermatis* observed in one strain of our study (MA1429) with a disk diffusion with no inhibition zone to FLZ.

The relationship between *ERG11* amino acid mutations and drug resistance has been reported in many pathogenic fungi such as *Aspergillus fumigatus*, *Candida* spp. and *Cryptococcus* spp. [58]. This is not the case of *M. pachydermatis*. Since now, few strains of *M. pachydermatis* have demonstrated to be resistant to azoles and only four amino acid mutations in *ERG11* have been described [23, 51, 52]. None of these substitutions have been confirmed to cause azole resistance using in vitro experiments and it’s not known which part of the protein is involved probably because there is no experimentally determined three-dimensional structure of the *ERG11* protein available. Based on the three-dimensional structure of *C. albicans ERG11*, the mutations G448D and G450D in *C. albicans* (synonymous with G459D and G461D in *M. pachydermatis*) are located near the heme-binding site and near the end of helix I of the protein, respectively [59, 60]. Also, mutation F145L (synonymous with F143S in *M. pachydermatis*) is located near the substrate channel [60]. Based on the three-dimensional structure of *C. neoformans* CYP51, residues A317 and F158 in *C. neoformans* (synonymous with A306S and F143S in *M. pachydermatis*, respectively) are in the heme binding site and mutation A313 in *C. neoformans* (A302T in *M. pachydermatis*) is part of one of the active site cavities [61]. However, the possibility that other deleterious substitutions, which are located outside of the active side, may also contribute to fungal resistance via other structural changes cannot be ruled out.

The presence or absence of amino acid substitutions may not be the solely cause of antifungal resistance [25, 55]. Other mechanisms of resistance against azoles have been suggested in *M. pachydermatis* as efflux pumps [14, 25]. Other possibility for these reduced susceptibilities is a chromosomal rearrangement that leads to an overexpression of the *ERG11* and *ERG4* genes. Kim et al. [29] found a tandemly quadruplicated region in chromosome 4 of two *M. pachydermatis* isolates with high in vitro MICs of KTZ. Thus, an overexpression of this region was observed that could be responsible of the higher MIC.

In conclusion, this study highlights the high diversity of sequences in *ERG11* gene, the primary target of azole antifungal drugs. This high diversity could be part of the high intrinsic variability of this gene in *M. pachydermatis*. Even though we found three mutations that were already reported, we also found some new mutations. Also, genotyping revealed small clusters of *ERG11*-mutate isolates. Although some mutant strains showed a reduced susceptibility to some antifungals, further studies would be necessary to completely understand the role of these mutations in the susceptibility against antifungal agents.

## Supplementary Information

Below is the link to the electronic supplementary material.

Supplementary file 1 (DOCX 32 kb)

Supplementary file 2 (DOCX 20 kb)
