# Supplementary material for: ERG11 Gene Variability and Azole Susceptibility in Malassezia pachydermatis
Source: Mycopathologia. 2022 Dec 10;188(1-2):21–34. doi: 10.1007/s11046-022-00696-9 (PMC10169892; doi:10.1007/s11046-022-00696-9)
Supplement: Supplementary file 1 — (DOCX 32 kb) [file 11046_2022_696_MOESM1_ESM.docx]

**Table S1.** Mean inhibition diameters of each strain included at 72h.

|  |  |  | **Mean inhibition diameters (mm) values at 72h** | | | | | | |
| --- | --- | --- | --- | --- | --- | --- | --- | --- | --- |
|  |  |  | **FLZ** | | **ITZ** | **KTZ** | | **AMB** | |
| **Strain** | **Species** | **Health status** | **Rosco**  **25µg/disk** | **Biorad**  **25µg/disk** | **Rosco**  **10µg/disk** | **Rosco**  **15µg/disk** | **Biorad**  **50µg/disk** | **Rosco**  **10µg/disk** | **Biorad**  **100µg/disk** |
| **Strains from healthy animals** | | | | | | | | | |
| CBS6535^a,b^ | Dog | Healthy | 26 | 36.5 | 34 | 50 | 55 | - | 19 |
| MA13^a,b^ | Dog | Healthy | 36 | 36 | 40 | 53 | 63 | - | 20 |
| MA52^a,b^ | Dog | Healthy | 34 | 37 | 38.5 | 51.5 | 58.5 | - | 20.5 |
| MA56^a,b^ | Dog | Healthy | 35 | 35.5 | 38 | 47.5 | 59.5 | - | 19 |
| MA94^a,b^ | Horse | Healthy | 31.5 | 39 | 34.5 | 49 | 53 | 17 | 20.5 |
| MA107*^b^ | Goat | Healthy | - | - | - | - | - | - | - |
| MA140^a,b^ | Cat | Healthy | 32.5 | 28 | 42 | 47 | 56 | 17 | 22 |
| MA475^a,b^ | Pig | Healthy | 29 | 30.5 | 29.5 | 44 | 53 | 17 | 20 |
| MA1595^b^ | Cow | Healthy | 36 | 39 | 35 | 49.5 | 57 | 15.5 | 18 |
| **Strains from animals with otitis** | | | | | | | | | |
| CBS1879^a,b^ | Dog | Otitis externa | 28 | 36.5 | 37.5 | 49 | 54.5 | 17.5 | 21 |
| CBS1884^a,b^ | Dog | Otitis externa | 35 | 42.5 | 42 | 51.5 | 69.5 | 18.5 | 24.5 |
| MA7^b^ | Dog | Recurrent otitis externa | 21 | 17.5 | 30 | 50 | 61 | 20.5 | 23 |
| MA8^b^ | Dog | Chronic otitis externa | 40.5 | 41.5 | 45.5 | 59 | 60 | 22 | 23 |
| MA10^b^ | Dog | Chronic otitis externa | 31 | 28 | 58 | 47 | 49 | 21 | 21.5 |
| MA138 | Dog | Recurrent otitis externa | 43.5 | 38 | 44 | 55 | 60 | 17 | 18.8 |
| MA165^a,b^ | Dog | Chronic otitis externa | 21 | 33.5 | 19 | 20 | 66.5 | 0 | 24.5 |
| MA173 | Dog | Otitis media | 31.5 | 35.5 | 37 | 48 | 56.5 | 18 | 20.5 |
| MA191 | Dog | Otitis externa | 7.5 | 6.5 | 35 | 52 | 66 | 18 | 20.5 |
| MA195^a,b^ | Dog | Otitis externa | 56.5 | 47 | 57 | 64 | 63.5 | 16 | 21 |
| MA259 | Dog | Chronic otitis externa | 33 | 39.5 | 34 | 49 | 62 | 19 | 21 |
| MA280^a,b^ | Dog | Chronic otitis externa | 24.5 | 9.5 | 46 | 62.5 | 64 | 15.5 | 19.5 |
| MA287 | Dog | Chronic otitis externa | 37 | 39 | 38.5 | 50 | 62 | 20 | 22.5 |
| MA312^a,b^ | Cat | Otitis externa | 36 | 38.5 | 37.5 | 48.5 | 62 | 19 | 21 |
| MA356^a,b^ | Dog | Otitis externa | 23.5 | 27.5 | 39 | 51 | 53.5 | 17.5 | 20.5 |
| MA361^b^ | Dog | Chronic otitis externa | 18 | 20 | 34 | 51 | 63 | 17 | 20 |
| MA396 | Dog | Purulent otitis externa | 23.5 | 20.5 | 40.5 | 59 | 70.5 | 19.5 | 22 |
| MA485 | Cat | Purulent otitis externa | 49 | 52 | 47.5 | 59 | 74 | 22 | 23 |
| MA506 | Dog | Chronic otitis externa | 32 | 26.5 | 34 | 52 | 57 | 18 | 23.5 |
| MA587^b^ | Cat | Otitis media | 37.5 | 54.5 | 36.5 | 46.5 | 38.5 | 13 | 17.5 |
| MA589 | Dog | Acute otitis externa | 19.5 | 27.5 | 46.5 | 54 | 68 | 19 | 23 |
| MA591 | Dog | Otitis media | 36.5 | 35 | 35 | 55.5 | 63 | 21 | 20 |
| MA641 | Dog | Acute otitis externa | 42 | 45.5 | 30 | 57 | 73 | 18 | 20 |
| MA646 | Dog | Chronic otitis externa | 29.5 | 21 | 42.5 | 62 | 65 | 16 | 18.5 |
| MA650 | Dog | Chronic otitis externa | 43 | 35 | 46 | 65.5 | 52 | 15 | 16.5 |
| MA672 | Dog | Otitis externa | 28.5 | 36 | 37 | 47 | 67 | 18 | 21 |
| MA680 | Dog | Otitis externa | 38 | 37.5 | 35 | 50 | 56.5 | 16.5 | 17.5 |
| MA688 | Dog | Chronic otitis externa | 36.5 | 38 | 44 | 57 | 59 | 19 | 23.5 |
| MA704 | Dog | Purulent otitis externa | 29 | 30 | 34.5 | 47 | 58.8 | 16 | 20 |
| MA762 | Dog | Chronic otitis externa | 45 | 40.5 | 44.5 | 57.8 | 56 | 15 | 15 |
| MA774 | Dog | Acute otitis externa | 25.5 | 40 | 33.5 | 42 | 59 | 18 | 17 |
| MA856^b^ | Dog | Acute otitis externa | 20 | 24 | 33 | 39.5 | 63.5 | 16.5 | 20.5 |
| MA860 | Dog | Acute otitis externa | 35.5 | 36.5 | 37 | 49 | 54.5 | 15 | 18.5 |
| MA872 | Cat | Chronic otitis externa | 42.5 | 41 | 41.5 | 55.5 | 60 | 14 | 18 |
| MA944^b^ | Dog | Chronic otitis externa | 15.5 | 27.5 | 29 | 45 | 59 | 16 | 21.5 |
| MA956 | Dog | Purulent otitis externa | 30 | 43 | 38 | 57 | 69.5 | 17 | 20 |
| MA968^b^ | Dog | Purulent otitis externa | 25 | 25 | 25 | 44.5 | 68.5 | 16 | 19 |
| MA972 | Dog | Acute otitis externa | 26.5 | 73 | 30 | 48 | 78 | 16 | 20.5 |
| MA1066 | Dog | Chronic otitis externa | 24 | 25.5 | 42.5 | 64 | 69 | 19 | 18 |
| MA1094 | Dog | Acute otitis externa | 40 | 43.5 | 55 | 73 | 69.5 | 20.5 | 22.5 |
| MA1134 | Dog | Chronic otitis externa | 17.5 | 6 | 38 | 56 | 66 | 18.5 | 22 |
| MA1142 | Dog | Acute otitis externa | 45.5 | 44 | 41 | 57 | 62 | 17.5 | 13 |
| MA1146 | Dog | Recurrent otitis externa | 35 | 37.5 | 37 | 52.5 | 60.5 | 14 | 17 |
| MA1158 | Dog | Acute otitis externa | 12.5 | 19 | 42.5 | 58.5 | 66.5 | 15.5 | 24 |
| MA1166 | Dog | Chronic otitis externa | 39 | 45.5 | 45 | 60 | 68 | 21 | 22 |
| MA1174 | Dog | Chronic otitis externa | 28 | 29.5 | 47 | 68.8 | 68 | 18.5 | 20.5 |
| MA1182 | Dog | Chronic otitis externa | 40 | 44 | 40 | 52.5 | 60 | 17 | 14.5 |
| MA1195 | Dog | Chronic otitis externa | 25 | 31 | 34 | 50 | 58 | 19.5 | 15 |
| MA1219 | Dog | Acute otitis externa | 39 | 40 | 38 | 55 | 61.5 | 18 | 14 |
| MA1239 | Dog | Chronic otitis externa | 34 | 38.5 | 36 | 45.5 | 57.5 | 16 | 16.5 |
| MA1243 | Dog | Chronic otitis externa | 37.5 | 39 | 42.5 | 58.5 | 65.5 | 19 | 21.5 |
| MA1257 | Dog | Chronic otitis externa | 34 | 40.5 | 41 | 54.5 | 63.5 | 22 | 23.5 |
| MA1305 | Dog | Chronic otitis externa | 18.5 | 15 | 39.5 | 56.5 | 65 | 18.5 | 19.5 |
| MA1382^a,b^ | Dog | Chronic otitis externa | 16 | 18.5 | 41 | 54 | 59.5 | 18.5 | 20 |
| MA1386 | Dog | Chronic otitis externa | 11 | 6.5 | 41 | 53 | 60 | 13.5 | 18 |
| MA1401^b^ | Dog | Recurrent otitis externa | 23 | 30 | 34 | 45 | 60 | 15 | 17 |
| MA1413 | Dog | Acute otitis externa | 30 | 26 | 52 | 65.5 | 69 | 18 | 19 |
| MA1417 | Dog | Acute otitis externa | 35 | 39.5 | 23.75 | 67 | 66 | 18.5 | 19.5 |
| MA1425 | Cat | Acute otitis externa | 28 | 32 | 35 | 46.5 | 59 | 17 | 19 |
| MA1437 | Dog | Purulent otitis externa | 40 | 45.5 | 42 | 58 | 65 | 15.5 | 20.5 |
| MA1441 | Dog | Purulent otitis externa | 45.5 | 43.5 | 43.5 | 62.5 | 60.5 | 14 | 18 |
| MA1466 | Dog | Acute otitis externa | 21 | 56.5 | 37.5 | 54 | 68 | 19 | 19 |
| MA1470 | Cat | Otitis media | 31 | 33.5 | 36.5 | 50 | 65 | 17.5 | 20 |
| MA1478^b^ | Dog | Acute otitis externa | 8 | 5.5 | 32 | 59 | 58.5 | 18 | 21.5 |
| MA1543 | Dog | Acute otitis externa | 26.5 | 27 | 43 | 58 | 64.5 | 18.5 | 17.5 |
| MA1544 | Dog | Acute otitis externa | 19 | 25.5 | 34.5 | 52.5 | 74.5 | 17 | 19.5 |
| MA1597 | Dog | Acute otitis externa | 42.5 | 52 | 43.5 | 59 | 74 | 16.5 | 16.5 |
| **Strains from animals with dermatitis** | | | | | | | | | |
| MA579^a,b^ | Cat | Dermatitis | 31.5 | 41 | 40 | 54 | 67 | 19 | 20 |
| MA593 | Cat | Dermatitis | 36 | 36 | 38.5 | 55 | 69.5 | 18.5 | 21.5 |
| MA597 | Cat | Dermatitis | 28 | 68.5 | 37.5 | 55 | 77 | 15 | 23 |
| MA601 | Cat | Dermatitis | 38 | 42 | 40 | 56 | 70.5 | 19 | 18.5 |
| MA615 | Cat | Dermatitis | 36.5 | 42 | 45 | 56 | 70.5 | 18 | 20 |
| MA794 | Dog | Dermatitis | 26.5 | 117.5 | 44 | 54 | 70 | 18 | 22 |
| MA1223 | Dog | Dermatitis | 42 | 45.5 | 42 | 54 | 68.5 | 20 | 18 |
| MA1231 | Dog | Dermatitis | 27.5 | 38.5 | 28 | 45.5 | 65 | 16.5 | 19 |
| MA1285 | Dog | Dermatitis | 39.5 | 39.5 | 43.5 | 59 | 65 | 20 | 19 |
| MA1289^b^ | Dog | Dermatitis | 19 | 19 | 40 | 52.5 | 65 | 16 | 16 |
| MA1398 | Dog | Dermatitis | 40 | 39.5 | 47.5 | 56.5 | 57.5 | 17 | 16.5 |
| MA1429^b^ | Dog | Dermatitis | 0 | 0 | 32.5 | 47 | 64.5 | 17.5 | 22 |
| MA1716^a,b^ | Dog | Dermatitis | - | 6.5 | - | - | 59 | - | 18 |

FLZ, fluconazole; ITZ, itraconazole; KTZ, ketoconazole; AMB, amphotericin B

^a^ Strains selected for E-test susceptibility assays.

^b^ Strains selected for multilocus and *ERG11* sequencing.

* Only DNA available.

- Not tested.
