# Supplementary material for: ERG11 Gene Variability and Azole Susceptibility in Malassezia pachydermatis
Source: Mycopathologia. 2022 Dec 10;188(1-2):21–34. doi: 10.1007/s11046-022-00696-9 (PMC10169892; doi:10.1007/s11046-022-00696-9)
Supplement: Supplementary file 2 — (DOCX 20 kb) [file 11046_2022_696_MOESM2_ESM.docx]

**Table S2.** Strains studied including original animal host, health status, and D1D2 rRNA, ITS rRNA, CHS2 and β-tubulin sequence types.

|  |  |  | ***Malassezia* genotype** | | | |
| --- | --- | --- | --- | --- | --- | --- |
| **Strain** | **Host** | **Health status** | **D1D2** | **ITS** | **CHS2** | **BTUB** |
| **Strains from healthy animals** | | | | | | |
| CBS6535^a^ | Dog | Healthy | I (AY743605) | I (AY743637) | II (KU313719) | I (KC573803) |
| MA13^a^ | Dog | Healthy | I | I | III (KU313720) | I |
| MA52^a^ | Dog | Healthy | I | II (KU313709) | I (EF140657) | II (KU313727) |
| MA56^a^ | Dog | Healthy | I | II | I | II |
| MA94^a^ | Horse | Healthy | I | III (KU313710) | I | I |
| MA107^a^ | Goat | Healthy | II (KU313705) | IV(KU313711) | IV (KU313721) | III (KU313728) |
| MA140^a^ | Cat | Healthy | I | V (KU313712) | V (KU313722) | IV (KU313729) |
| MA475^a^ | Pig | Healthy | II | IX (KU313716) | VIII (KU313725) | VII (KU313732) |
| MA1595^b^ | Cow | Healthy | I | III | I | I |
| **Strains from animals with otitis** | | | | | | |
| CBS1879^a^ | Dog | Otitis externa | I | I | I | I |
| CBS1884^a^ | Dog | Otitis externa | I | I | I | II |
| MA7^b^ | Dog | Recurrent otitis externa | V (KU313708) | XIV (ON791562) | IX (KU313726) | VIII (KU313733) |
| MA8^b^ | Dog | Chronic otitis externa | V | XII (KY655279) | IX | VIII |
| MA10^b^ | Dog | Chronic otitis externa | IV (KU313707) | XV (ON791563) | V | X (ON814675) |
| MA165^b^ | Dog | Chronic otitis externa | V | XVII (ON791564) | IX | VIII |
| MA195^a^ | Dog | Acute otitis externa | I | VI (KU313713) | I | I |
| MA280^a^ | Dog | Chronic otitis externa | III (KU313706) | VII (KU313714) | IV | III |
| MA312^a^ | Cat | Otitis externa | IV | VIII (KU313715) | VI (KU313723) | V (KU313730) |
| MA356^a^ | Dog | Otitis externa | III | IV | VII | VI (KU313731) |
| MA361^b^ | Dog | Chronic otitis externa | III | XVI (ON791565) | IV | III |
| MA587^a^ | Cat | Otitis media | I | I | I | XI (ON814676) |
| MA856^a^ | Dog | Acute otitis externa | V | XIV | IX | VIII |
| MA944^b^ | Dog | Chronic otitis externa | V | XIV | IX | VIII |
| MA968^b^ | Dog | Purulent otitis externa | V | XIV | IX | VIII |
| MA1382^a^ | Dog | Chronic otitis externa | V | XI (KU313718) | IX | VIII |
| MA1401^b^ | Dog | Recurrent otitis externa | VI (ON787824) | III | I | II |
| MA1478^b^ | Dog | Acute otitis externa | III | XVI | IV | III |
| **Strains from animals with dermatitis** | | | | | | |
| MA579^a^ | Cat | Dermatitis | IV | X (KU313717) | V | IV |
| MA1289^b^ | Dog | Dermatitis | III | XVI | IV | III |
| MA1429^b^ | Dog | Dermatitis | III | XVI | VII (KU313724) | VIII |
| MA1716^b^ | Dog | Dermatitis | I | I | I | I |

^a^ Sequence types determined in a previous study

^b^ Sequence types determined in the present study.
